# Asexual thalli originated from sporophytic thalli via apomeiosis in the green seaweed *Ulva*

**DOI:** 10.1038/s41598-019-50070-x

**Published:** 2019-09-18

**Authors:** Kensuke Ichihara, Tomokazu Yamazaki, Shinichi Miyamura, Masanori Hiraoka, Shigeyuki Kawano

**Affiliations:** 1 Department of Integrated Biosciences, Graduate School of Frontier Sciences, The University of Tokyo, Kashiwanoha 5-1-5, Kashiwa, Chiba 277-8562 Japan; 2 Faculty of Life and Environmental Sciences, University of Tsukuba, Tsukuba, Ibaraki 305-8572 Japan; 3 Usa Marine Biological Institute, Kochi University, Inoshiri 194, Usa, Tosa, Kochi 781-1164 Japan; 4 Present Address: Field Science Center for Northern Biosphere, Hokkaido University, 1-133-31, Funami-Cho, Muroran, 051-0013 Japan; 5 Present Address: Future Center Initiative, The University of Tokyo, Wakashiba, Kashiwa, Chiba, 277-0871 Japan

**Keywords:** Meiosis, Chromosomes, Plant reproduction

## Abstract

Apomixis is an asexual reproduction system without fertilization, which is an important proliferation strategy for plants and algae. Here, we report on the apomeiosis in the green seaweed *Ulva prolifera*, which has sexual and obligate asexual populations. Genomic PCR of mating type (MT)-locus genes revealed asexual thalli carrying both MT genomes. Observation of the chromosomes during the formation of each type of reproductive cell revealed that cells in asexual thalli performed apomeiosis without chromosome reduction. Moreover, genotyping revealed that laboratory-cultured sporophytic thalli produced not only each type of gametophyte but also diploid thalli carrying the mt^−^ and mt^+^ genome (mt^±^ thallus strains). The mt^±^ thallus strain released diploid biflagellate zoids, with ultrastructure and behavior similar to mt^+^ gametes. Additionally, a transcriptomic analysis revealed that some meiosis-related genes (*Mei2L* and *RAD1*) were highly expressed in the quadriflagellate zoosporoids. Our results strongly suggest that asexual thalli originally evolved via apomeiosis in sporophytic thalli.

## Introduction

Plants, including flowering plants, ferns, and bryophytes, produce their progenies via sexual or asexual reproduction systems. Apomixis is an asexual reproduction in which mother plants produce genetically identical progenies in sexual organs without fertilization. The process of apomictic seed development in land plants consists of three distinct steps: apomeiosis (avoidance of meiosis), parthenogenesis (development of an embryo without fertilization), and functional endosperm development^1,2^. The elucidation of the mechanism of apomixis is important not only for biological interests, but also for agricultural technology, because it may be possible to add this trait to crops^3,4^.

The green seaweed *Ulva* belongs to the class Ulvophyceae and primarily exists in marine habitats, but is also found in brackish water and even in freshwater^5–9^. In the lifecycle of *Ulva*, gametophytes of two mating types – minus (mt^−^) and plus (mt^+^) – release biflagellate gametes with positive phototaxis; mt^−^ gametes then conjugate with mt^+^ gametes and vice versa; the zygote develops into the sporophytic phase^10^. Gametes that fail to conjugate develop parthenogenetically into gametophytes^9^. Sporophytes generate and release quadriflagellate meiospores through meiosis, which then develop into genetically separate mt^−^ or mt^+^ gametophytes^9^ (Supplementary Fig. 1). Thus, each sexual individual has a lifecycle that consists of a gametophytic stage and a sporophytic stage^9^.

Instead of undergoing the ordinary sexual lifecycle, several *Ulva* species have obligately asexual lifecycles that occur without sexual reproduction via meiosis and conjugation. Sexual and asexual populations coexist in phylogenetically independent lineages, suggesting that each asexual population evolved separately in each lineage^11–13^. There are two types of asexual cycles, one via biflagellate zoids, and the other via quadriflagellate zoids (Supplementary Fig. 1)^14^. Both biflagellate and quadriflagellate zoids develop directly into asexual thalli. The asexual zoids are termed ‘zoosporoids’^14^. Like other *Ulva* species, *Ulva prolifera* has both types of asexual populations, and the amount of DNA in the cells of an asexual thallus in this species is similar to that in the cells the sporophytic thallus^11^. Although previous research has indicated that asexual thalli are diploid, it has not been examined whether each asexual thallus was generated by diploidization of the gametophyte or by apomixis in the sporophyte.

In the 2010s, macroalgal genomics became an active area of research, and the genomes of some brown and red marine alga have been sequenced^15–17^. The genome of the model brown alga *Ectocarpus* was released in 2010^15^ and was recently improved by deep sequencing using next-generation sequencing technology^18^. Sex-determining regions (SDRs) of *Ectocarpus* were also identified^19^, and a candidate male reproductive gene, which is similar to a receptor involved in sperm-egg recognition in sea urchins, was found by RNA sequencing (RNA-Seq) analysis of mature male and female gametophytes^20^. Although genomic analysis in Ulvophyceae has lagged behind that of other taxa, the genome of *Ulva mutabilis* has recently been published^21^. Our recent analysis of the *Ulva partita* genome also revealed a mating-type (MT) locus^22^. The MT locus contains MT-specific genes and gametologs that are shared, but with differentiated sequences, between the two MTs. One of the gametologs, the *PRA1* (proliferation-associated protein 1) gene, has been isolated from individual MT strains of other *Ulva* species, and the molecular phylogenetic tree of the *Ulva* species reveals that these isolated genes are clearly classified into two distinct clades associated with the MTs, which suggests that the MT locus is conserved in the genus *Ulva* and evolved independently within the MTs of each species. The availability of genomic data and the discovery of the MT locus now provide a molecular basis for studying the origin and evolution of sex, the sex-determination system, and the pathway by which asexual populations emerged in the Ulvophyceae.

In this study, we explored the evolutionary process by which asexual thalli (biflagellate type and quadriflagellate type) arose from the sexual population in the green seaweed *Ulva*. We used MT locus information and DNA content analysis to determine whether each asexual thallus was produced by diploidization of the gametophyte or by apomixis in the sporophyte. We also observed chromosomal dynamics during reproductive cell formation to investigate the nuclear division pattern in the cells. Finally, we performed RNA-Seq analysis to search for the causative genes for apomixis.

## Results

### Genotyping of culture strains using genes in the MT locus

**Figure 1:**
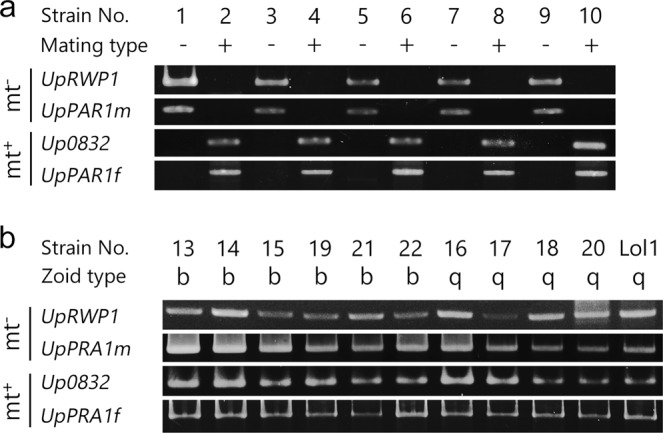
Genomic PCR of the MT-locus gene homologs for distinct culture strains. The presence of orthologous genes of the *U*. *partita* MT-locus genes (mt^−^ genes: *UpRWP1*, *UpPAR1m*, mt^+^ genes: *Up*00832, *UpPAR1f*) was confirmed in 21 strains (10 gametophytic thallus strains and 11 asexual thallus strains) isolated from different areas along the Japanese coast and Denmark: (**a**) gametophytic thallus strains, (**b**) asexual thallus strains. Details of each strain are summarized in Table 1.

Linkages between *Ulva* MT locus–specific marker genes and each MT of *U*. *prolifera* were examined in culture strains established from thalli collected in various regions in Japan and Denmark (Table 1). Two mt^−^ MT locus–specific markers were detected in all mt^−^ gametophytic thalli, and two mt^+^ MT locus–specific markers were detected in all mt^+^ gametophytic thalli. Each MT locus–specific marker was not detected in gametophytic thalli of the opposite MT. Thus, the MT locus–specific marker genes were completely linked to the MT of each gametophytic thallus (Fig. 1a). Conversely, both MT locus–specific markers were detected in each strain of asexual thallus (Fig. 1b), suggesting that the asexual thalli produced via a biflagellate zoosporoid and the asexual thalli produced via a quadriflagellate zoosporoid are carrying both the mt^−^ and mt^+^ MT loci.

**Table 1:** List of strains of *U*. *prolifera* examined in this study.

Type of thallus	Strain name	Mating type	Collection date	Location	Accession No.				
					*RWP1*	*PAR1m*	*00832*	*PAR1f*	reference
Gametophytic thallus	Up01^a^	mt^−^	Feb. 25, 2001	S1, Shimanto River, Kochi Pref., Japan^37^	LC480318	LC4803120	n.d.	n.d.	^6^
	Up02^b^	mt^+^	Feb. 25, 2001	S1, Shimanto River, Kochi Pref., Japan^37^	n.d.	n.d.	LC480316	LC480322	^6^
	Up03	mt^−^	June 1, 2008	Nobusha River, Mashike Hokkaido, Japan	LC480319	LC4803121	n.d.	n.d.	—
	Up04	mt^+^	June 1, 2008	Nobusha River, Mashike Hokkaido, Japan	n.d.	n.d.	LC480317	LC480323	—
	Up05	mt^−^	Mar. 11, 2012	Nakayama River, Saijo city, Ehime Pref., Japan	—	—	n.d.	n.d.	—
	Up06	mt^+^	Mar. 11, 2012	Nakayama River, Saijo city, Ehime Pref., Japan	n.d.	n.d.	—	—	—
	Up07	mt^−^	Mar. 11, 2012	Kamo River, Saijo city, Ehime Pref., Japan	—	—	n.d.	n.d.	—
	Up08	mt^+^	Mar. 11, 2012	Kamo River, Saijo city, Ehime Pref., Japan	n.d.	n.d.	—	—	—
	Up09	mt^−^	Dec. 16, 2012	Adake River, Nakatane Cho, Kagoshima Pref., Japan	—	—	n.d.	n.d.	—
	Up10	mt^+^	Dec. 16, 2012	Adake River, Nakatane Cho, Kagoshima Pref., Japan	n.d.	n.d.	—	—	—
Asexual thallus via a biflagellate zoosporoid	Up13^d^	—	Mar. 1, 2009	S4, Shimanto River, Kochi Pref., Japan^37^	LC480318	LC4803120	LC480316	LC480322	—
	Up14	—	Mar. 1, 2009	S4, Shimanto River, Kochi Pref., Japan^37^	LC480318	LC4803120	LC480316	LC480322	—
	Up15^b^	—	Mar. 2, 2010	Takeshima River, Kochi Pref., Japan	—	—	—	—	—
	Up19	—	June 18, 2012	Naka River, Matsuzaki, Shizuoka Pref., Japan	—	—	—	—	—
	Up21	—	Dec. 10, 2010	Ohta River, Hiroshima city, Hiroshima pref., Japan	—	—	—	—	—
	Up22	—	May 23, 2001	Yoshino River, Aizumi, Tokushima Pref., Japan	LC480318	LC4803120	LC480317	LC480326	—
	SH01^b^	—	June 2, 2015	Otonashi River, Kamakura City, Kanagawa Pref., Japan	—	—	—	—	—
	SH05^b^	—	June 2, 2015	Otonashi River, Kamakura City, Kanagawa Pref., Japan	LC480318	LC4803120	LC480316	LC480325	—
Asexual thallus via a quadriflagellate zoosporoid	Up16	—	Mar. 1, 2009	S4, Shimanto River, Kochi Pref., Japan^37^	LC480318	LC4803120	LC480316	LC480322	—
	Up17^a^	—	Mar. 2, 2010	Takeshima River, Kochi Pref., Japan	LC480318	LC4803120	LC480316	LC480324	—
	Up18	—	June 18, 2012	Naka River, Matsuzaki, Shizuoka Pref., Japan	—	—	—	—	—
	Up20	—	July 16, 2012	Kushiro River, Kushiro City, Hokkaido, Japan	—	—	—	—	—
	D1	—	Aug. 14, 2010	Nebbelunde, Denmark (type locality)	—	—	—	—	^49^
Sporophytic thallus	Up01 x Up02^c^	—							—
Mt^±^ thallus developed from meiospores released by Up01 x Up02	F1-3^b^	—							—
	F1-14^d^	—							—
	F1-17^b^	—							—

^a^Indicates strains used in DNA content analysis, FE-SEM, observation of chromosomes and RNA-Seq analysis. ^b^Indicates strains used in FE-SEM. ^c^Indicates a strain used in observation of chromosomes. ^d^Indicates a strain used in DNA content analysis and FE-SEM.

### Comparing the DNA content of gametes, biflagellate zoosporoids, and quadriflagellate zoosporoids

**Figure 2:**
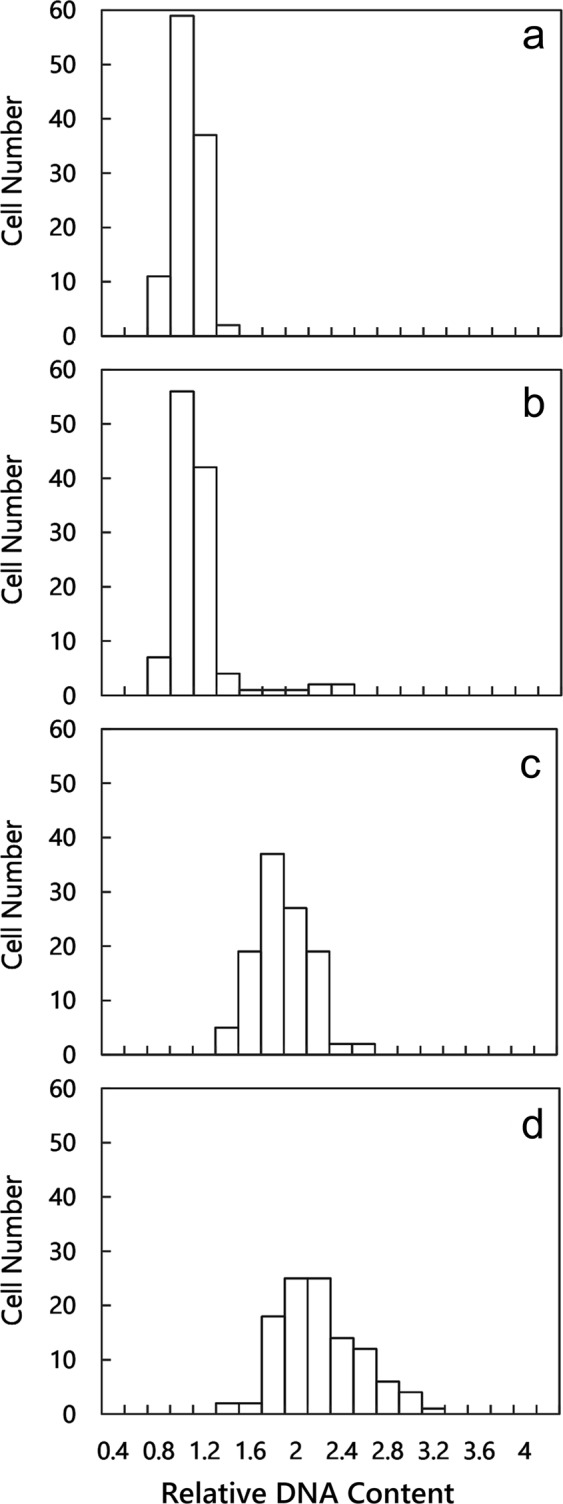
Histogram comparing the relative nuclear DNA content among gametes and zoosporoids: (**a**) mt^−^ gametes released by Up01 gametophytic thallus, (**b**) mt^+^ gametes released by Up02 gametophytic thallus, (**c**) biflagellate zoosporoids released by Up13 asexual thallus, (**d**) quadriflagellate zoosporoids released by Up17 asexual thallus.

The nuclear DNA contents of mt^−^ gametes, mt^+^ gametes, biflagellate zoosporoids, and quadriflagellate zoosporoids were measured by fluorescence microscopy for the determination of the ploidy. After calibrating the fluorescence of nuclear DNA using the fluorescence of standard beads, the nuclear DNA content of each reproductive cell was measured. The relative nuclear DNA content of the mt^−^ gametes was consistent with that of mt^+^ gametes, and each of the values were designated 1 C (Fig. 2a,b). Comparison of the DNA contents of gametes with those of the biflagellate or quadriflagellate zoosporoids yielded the estimate that the DNA content of the biflagellate and quadriflagellate zoosporoids were 2 C (Fig. 2c,d). These results indicate that the two types of zoosporoids are cytologically diploids.

### Morphological observation of gametes, biflagellate zoosporoids, and quadriflagellate zoosporoids by field-emission scanning electron microscopy (FE-SEM)

**Figure 3:**
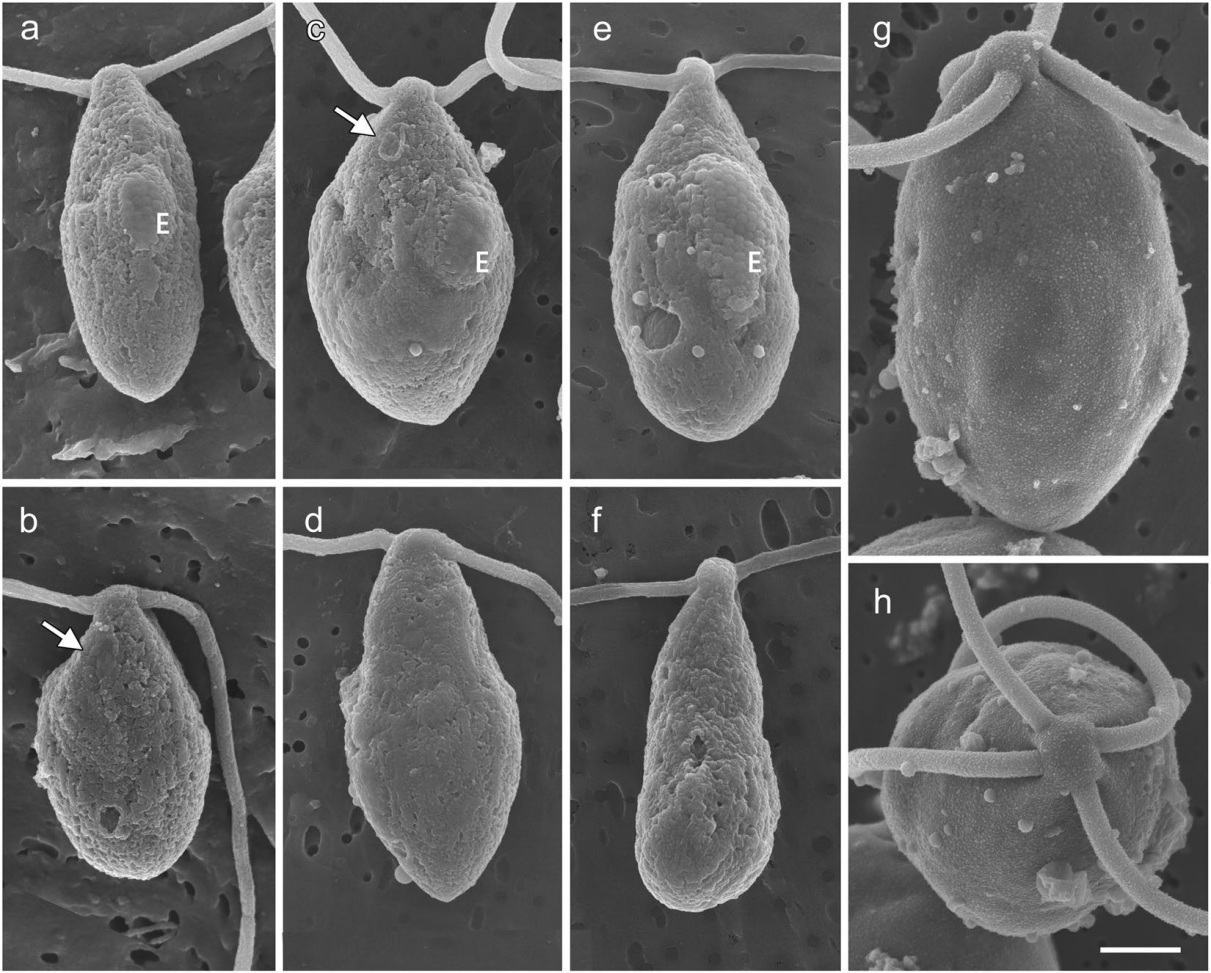
Field emission scanning electron micrographs of gametes and zoosporoids of *Ulva prolifera:* (**a**,**b**) mt^−^ gametes released by Up01 gametophytic thallus, (**c**,**d**) mt^+^ gametes released by Up02 gametophytic thallus, (**e**,**f**) biflagellate zoosporoids released by Up15 asexual thallus, (**g**,**h**) quadriflagellate zoosporoids released by Up17 asexual thallus. Arrow indicates the mating structure. E, eyespot. Scale bar, 1 μm.

The mt^−^ and mt^+^ gametes, the biflagellate zoosporoids, and the quadriflagellate zoosporoids were observed by FE-SEM to determine their morphological differences. The mt^−^ gametes had a mating structure (i.e., cell fusion apparatus) on the opposite side of the eyespot (Fig. 3a,b). In contrast, the mating structure of the mt^+^ gamete was located on the same side of the eyespot (Fig. 3c,d). The biflagellate zoosporoids had a larger eyespot than those of the gametes (Fig. 3e,f), and no mating structure was found on either the same or opposite side from the eyespot. The quadriflagellate zoosporoids without a mating structure had the largest cell size and had four cruciately arranged flagella (Fig. 3g,h).

### Observation of chromosomal dynamics during the formation of reproductive cells

**Figure 4:**
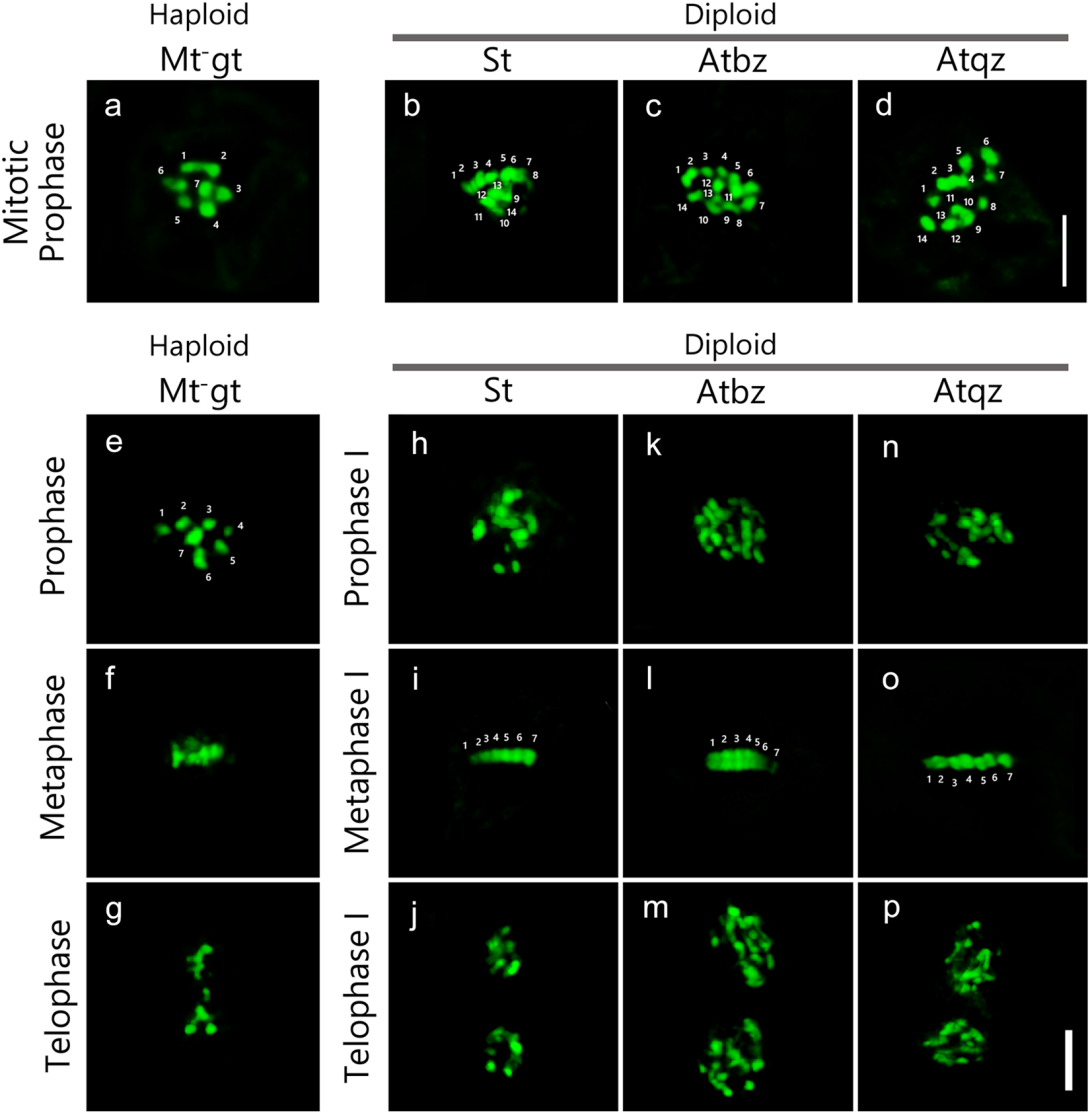
Chromosome dynamics during mitosis and each reproductive cell formation. (**a–d**) Chromosome dynamics in mitosis: (**a**) an mt^−^ gametophytic thallus (Up01), (**b**) a sporophytic thallus (Up01 × Up02), (**c**) an asexual thallus produced via a biflagellate zoosporoid (Up13), (**d**) an asexual thallus produced via a quadriflagellate zoosporoid (Up17). Seven chromosomes were observed in a gametophytic thallus (a) and 14 chromosomes were observed in a sporophytic thallus and asexual thalli (**b–d**). (**e–q**) Chromosome dynamics during the formation of each reproductive cell. (**e–g**) Mitotic nuclear division for gamete formation in an mt^−^ gametophytic thallus (Up01). (**h–q**) Meiotic or apomeiotic nuclear division for meiospores or zoosporoids formation. (**h–j**) A sporophytic thallus (Up01 × Up02). (**k–m**) An asexual thallus produced via a biflagellate zoosporoid (Up13); (**n–p**) an asexual thallus produced via a quadriflagellate zoosporoid (Up17). Seven bivalent chromosomes were observed during meta-phase of a sporophytic thallus and two types of an asexual thallus. Mt^−^gt, mt^−^ gametophytic thallus; St, sporophytic thallus; Atbz, asexual thallus produced via a biflagellate zoosporoid; Atqz, asexual thallus produced via a quadriflagellate zoosporoid. Scale bar, 3 μm.

To understand the mechanism of gamete, meiospore, and diploid zoosporoid formation, the chromosomal dynamics were observed during each stage of reproductive cell formation. First, the cells of the mt^−^ gametophytic thallus during mitotic prophase were observed to reveal the chromosome number of the gametophyte, and seven chromosomes were clearly observed (Fig. 4a). Fourteen chromosomes were observed during the mitotic prophase in cells of the sporophytic thallus; those of the asexual thallus produced via biflagellate zoosporoids and those of the asexual thallus produced via quadriflagellate zoosporoids (Fig. 4b–d). Additionally, 14 chromosomes were located on the equatorial plate in cells of the asexual thallus produced via quadriflagellate zoosporoids during mitotic mid-metaphase (Supplementary Fig. 2).

During the first nuclear division before gamete formation, seven chromosomes were found in cells of the mt^−^ gametophytic thallus (Fig. 4e). In metaphase, the seven chromosomes were arranged at the equatorial plate (Fig. 4f), and then the homologous chromosomes were segregated toward opposite poles (Fig. 4g). In cells of the sporophytic thallus, the chromosomes were entangled in meiotic prophase I (Fig. 4h). The seven bivalent chromosomes were arranged at the equatorial plate in meiotic metaphase I (Fig. 4i), and the seven chromosomes were observed at both polar areas after the first meiotic division (Fig. 4j). The chromosome number reduced from 14 to 7 in this first meiotic phase. In the cells of each asexual thallus produced via biflagellate zoosporoids and quadriflagellate zoosporoids, entangled chromosomes were observed in prophase I (Fig. 4k,n). In metaphase I, the seven bivalent chromosomes aligned at the equatorial plate (Fig. 4l,o), and after the segregation of chromosomes toward opposite poles, the number of chromosomes was unreduced (Fig. 4m,p). The reduction in the chromosome number was not observed in the cells of the two types of asexual thalli in this first meiotic phase.

**Figure 5:**
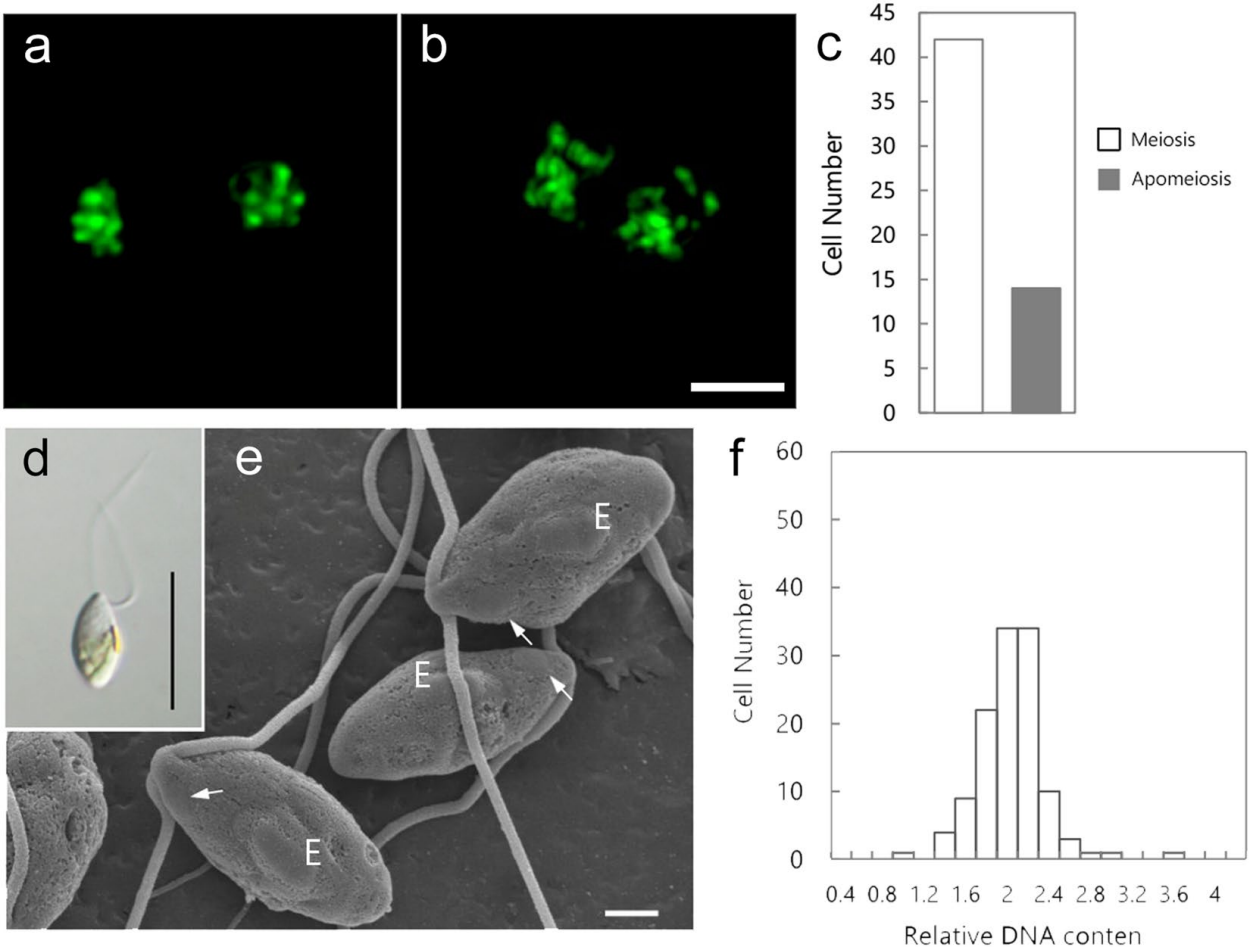
Apomeiosis in the cells of a sporophytic thallus (Up01 × Up02) and observation of diploid biflagellate zoids released from the mt^±^ thallus. (**a**) Meiotic telophase I in normal meiosis. (**b**) Meiotic telophase I in apomeiosis; chromosome numbers were not reduced. (**c**) Cells number of progressing meiosis or apomeiosis in a sporophytic thallus. n = 56. (**d**,**e**) Morphology of biflagellate zoids released from the mt^±^ thallus. (**d**) Light micrograph. (**e**) Field-emission scanning electron micrograph. Arrow indicates the mating structure. E, eyespot. (**f**) Histogram of relative nuclear DNA content in diploid biflagellate zoids. Scale bar, 3 μm (**a**,**b**), 10 μm (**d**), 1 μm (**e)**.

The apomeiotic process, which was similar to the phenomena that occurred in both of the asexual thalli, was also observed in the cells of the sporophytic thallus. Normal first meiotic division was observed in 42 cells, whereas apomeiotic division was observed in 14 cells (n = 56; Fig. 5a–c). In the latter case, the reduction in the chromosome number was not observed, and the 14 chromosomes existed in telophase I (Fig. 5b). To confirm that the apomeiotic process occurred in the sporophytic thallus, the genotypes of 66 thalli that developed from meiospores were examined using the above MT locus–specific markers. Genotyping revealed that 59 thalli were haploid gametophytic thalli (mt^−^: 19, mt^+^: 40). However, both of the MT markers were detected in seven thalli (which were defined as the mt^±^ thallus strain). This result also provided evidence for the occurrence of apomeiosis during reproductive cell formation in sporophytic thalli. Additionally, the biflagellate zoids released from the mt^±^ thalli (F1-3, F1-7, F1-14) had the round mating structure on the same side as the eyespot (Fig. 5d,e) and the amount of DNA in the biflagellate zoids was 2 C, which was the same as that of the biflagellate or quadriflagellate zoosporoids (Fig. 5f). The biflagellate zoids released from the F1-14 strain were crossed with mt^−^ or mt^+^ gametes, resulting in conjugation between the biflagellate zoids and the mt^−^ gametes, but not the mt^+^ gametes (Supplementary Fig. 3).

### Expression of the MT-locus genes and meiosis-related genes in reproductive cells

**Figure 6:**
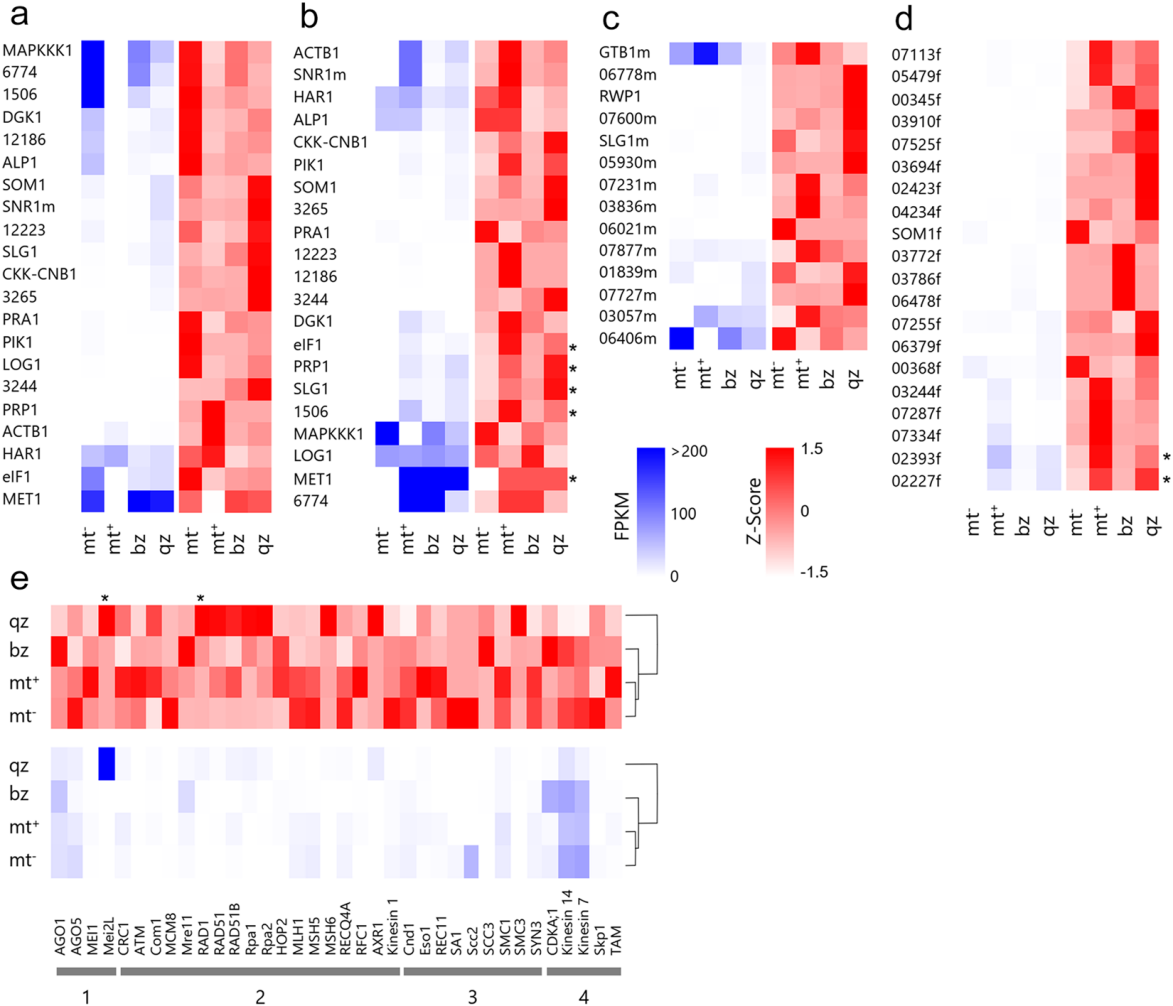
Changes in the expression of the mating type (MT) locus homologous genes and the meiosis-related genes in gametes and zoosporoids: (**a)** mt^−^ gametologs, (**b**) mt^+^ gametologs, (**c**) mt^−^-specific genes, (**d**) mt^+^-specific genes, (**e**) meiosis-related genes. Numbers under the gene names in (**e**) indicate the meiotic process category: 1. entry into meiosis, 2. recombination, 3. sister chromatid cohesion, 4. cell cycle control: spindle–cytokinesis. The blue heatmap shows the fragments per kilobase of exon per million fragments (FPKM) values. High FPKM values (>200) are shown in the same color as values of 200. The red heat map shows z-score calculated from FPKM values. Differentially expressed genes are indicated by an asterisk on the right side of or above the heat maps (*p < 0.05). mt^−^, mt^−^ gamete; mt^+^, mt^+^ gamete; bz, biflagellate zoosporoid; qz, quadriflagellate zoosporoid.

To understand the molecular basis of the morphological, cytological, and genetic differences between the reproductive cells, RNA-Seq analysis was conducted (Supplementary Table 1); this focused on the MT-locus genes and the meiosis-related genes. Genes that were homologous to the MT-locus genes of *U*. *partita* were searched using BLASTX querying a database of *U*. *prolifera* contigs assembled by RNA-Seq, and their expression levels were estimated (Fig. 6). 60% of the mt^−^ specific genes (14/23), 45% of the mt^+^ specific genes (20/44), and 91% of the gametologs (21/23) were found to be homologous to the contigs of *U*. *prolifera* (Supplementary Tables 2 and 3). Many gametologs were highly expressed in the gametes of the individual MTs (Fig. 6a,b). All sets of gametologs derived from both MTs were expressed in the biflagellate and quadriflagellate zoosporoids, and the expression patterns of gametologs were clearly different when comparing both gametes and zoosporoids (Fig. 6a,b; Supplementary Tables 4 and 5). The expression pattern of MT-specific genes did not show a distinct tendency when comparing the gametes and zoosporoids (Supplementary Tables 6 and 7). Differentially expressed genes (DEGs) were not detected among mt^−^ gametologs or mt^-^ specific genes, while there were some DEGs in mt^+^ gametologs (*elF1*, *PRP1*, *SLG1*, *01506* and *MET1*) and in mt^+^ specific genes (*02393 f* and *02227 f*).

Thirty-six meiosis-related genes from land plants were found in the *U*. *prolifera* RNA-Seq contigs (Supplementary Table 8). The expression level of each homologous gene is shown as a heatmap in Fig. 6e. Clustering analysis revealed that the gene expression tendencies of the mt^−^ and mt^+^ gametes were similar, and that those of the quadriflagellate zoosporoids and the mt^+^ gametes were markedly different (Fig. 6e; Supplementary Table 9). The expression of *Mei2L* (Category 1) and *RAD1* (Category 2) genes differed significantly between each type of reproductive cell.

## Discussion

This study revealed that the asexual thalli of *Ulva prolifera* are diploid and have the genes of both of the MT loci, regardless of the number of flagella on the zoosporoids. Although the diploidy of asexual thalli has previously been suggested^11^, the cytological, DNA content, chromosome number, and genotype results presented here strongly suggest that the asexual thalli contain both the mt^−^ and mt^+^ genomes and originated from sporophytes.

The mating structure at the tip of each gamete was observed and these structures corresponded to previous findings^23^. The conclusion that the function of the mating structure in the Ulvophyceae is for fertilization was also supported by the localization of membrane fusion protein HAP2/GCS1^24^. Conversely, in the biflagellate zoosporoids, no mating structure could be found on the eyespot side or the side opposite from the eyespot, even in the samples collected in the field or from isolated cultures. The biflagellate zoosporoids were completely lacking in the mating structure and the ability to conjugate, although this study did not elucidate the mechanism by which this occurred. The diploid biflagellate zoids with a mating structure on the same side as the eyespots (Fig. 5e) released from the diploid mt^±^ thalli were obtained from a sporophytic thallus and conjugated with mt^−^ gametes (Supplementary Fig. 3). These phenotypes indicated that mt^+^ is dominant over mt^−^ in *Ulva*, which is contrary to the results for *Chlamydomonas*. In *Chlamydomonas*, heterozygous diploid cells displayed the characteristics of mt^−^, indicating that mt^−^ is dominant over mt^+^ ^25^. A discrepancy between the Ulvophyceae and *Chlamydomonas* in the relationship between sex and cell fusion site has been also reported^23,26,27^. This incongruence between two green algal lineages may be the result of differences in the genetic pathways that determine MT.

In this study, we observed chromosome dynamics during the formation of reproductive cells in the green algae *U*. *prolifera*. Observation by fluorescent microscopy revealed that normal meiosis I occurred in a major portion of the cells in the sporophytes, but some of the sporophyte cells, and cells of the asexual thalli produced via biflagellate and quadriflagellate zoosporoids, carried out apomeiosis without a reduction in the number of chromosomes (Supplementary Fig. 4). Bivalent chromosomes were clearly observed during the formation of biflagellate and quadriflagellate zoosporoids, indicating that cells in asexual thalli begin the meiosis I process, but do not complete it. These chromosomal dynamics indicate that zoosporoid formation in asexual thalli does not occur via somatic cells like the mitotic process, but rather by the apomeiotic process. The observed pattern of apomeiosis, in which bivalent chromosomes are formed, but sister chromatids segregate in the first division, is similar to the nuclear division pattern observed in the *Arabidopsis MiMe* (*osd1/Atspo11-1/Atrec8*) mutant^28^. In the *MiMe* mutant, *Atspo11-1* and *Atrec8* mutations instigate a mitotic-like first meiotic division, while an *osd1* mutation prevents the second meiotic division. In the *U*. *prolifera* asexual thallus, it is unclear that the second meiotic division is skipped (as it is in the *MiMe* mutant); understanding the whole meiotic or apomeiotic process that occurs in each reproductive cell would require flowcytometric analysis for measuring ploidy before cells begin the meiotic division process. Meiosis without a decrease in the number of the chromosomes in the first meiotic division has been also reported in land plants having holocentromeric chromosomes^29,30^. There are no reports of holocentromeres in the Ulvophyceae, and thus a more detailed analysis of centromere structure in this group would also help to elucidate the meiosis process in *U*. *prolifera*.

Apomictic plants can be produced through outcrossing between genetically distant strains in the rhodophyta *Caloglossa*^31^. In *U*. *prolifera*, some asexual thalli were heterogeneous in an exon region of *Hsp90*, a single-copy gene, and a similar process may contribute to the generation of asexual thalli in *Ulva*^13^. In this study, the Up01 × Up02 sporophyte was found to produce asexual thalli through the apomeiotic process (Fig. 5; Supplementary Fig. 5). However, the Up01 and Up02 strains were isolated from meiospores released by a single sporophyte (E21)^6,32^. This result suggests that apomeiosis may occur readily in the sporophytic thallus of *U*. *prolifera* regardless of the genetic distance between male and female gametes. In *U*. *mutabilis*, sporophytic thalli rarely released diploid quadriflagellate zoids (0.5%), which grow into mt^−^/mt^−^, mt^+^/mt^+^, or mt^+^/mt^−^ thalli^10^. In comparison, our results revealed a high production rate of mt^±^ (mt^+^/mt^−^) thalli. This difference may be due to species characteristics or experimental conditions. The proportion of normal embryos and embryos produced by apomixis varies in land plants depending on environmental stressors^33,34^. In the alpine plant *Ranunculus kuepferi*, which is a facultative apomict, cold stress increases the frequency of apomictic seed formation^35^. These results indicate that environmental stresses affect the determination of the reproductive mode in plants. The gametophytes and sporophytes used in the present study were originally collected from a river estuarine area with low salinity^6^. Our experiment was carried out under seawater conditions, which might have induced a high rate of apomeiosis in the sporophytes due to salinity stress (Fig. 5).

Two mt^+^-specific genes were slightly up-regulated in mt^+^ gametes and quadriflagellate zoosporoids, but these genes show no homology to known proteins. Some gametologs (e.g., mt^+^ *MAPKKK1* and mt^−^ *HAR1*) and MT-specific genes (*GTB1m*1 and *00368f1*) were expressed in one MT gamete but not in the opposite one (Fig. 6a,b). This finding suggests either that these genes have already been translocated from the MT locus in *U*. *prolifera* to an autosomal region or that the *U*. *partita* homologous gene was translocated from the autosome after the two species diverged from each other. The observation of chromosome dynamics during reproductive cell formation indicated that biflagellate and quadriflagellate zoosporoids were produced through the meiotic process rather than the mitotic process. The *Mei2*-*Like* gene homolog and *RAD1* homolog were especially highly expressed in quadriflagellate zoosporoids. Expression and mutant analysis of the *AML* (*Arabidopsis*-mei2-like) gene in *Arabidopsis thaliana* suggests that *AML* is involved in meiosis as well as vegetative growth, indicating that *Mei2* homologs are meiosis-related genes that are widely conserved from yeasts to land plants^36^. High levels of expression of the *Mei2-Like* (*Mei2L*) homolog in quadriflagellate zoosporoids suggests that the *Mei2L* homolog has a function during meiosis in *Ulva* (Fig. 6e).

Regardless of the number of flagella, each asexual thallus has both mt^−^ and mt^+^ genomes, thus, each asexual thallus was originally produced through apomixis via apomeiosis in a sporophytic thallus (Supplementary Fig. 4). Transcriptome analysis revealed that the MT-locus genes were expressed in the biflagellate and quadriflagellate zoosporoids, and the *Mei2L* homolog was upregulated in the quadriflagellate zoosporoids. In the next step, transcriptomic analysis will be conducted during the meiotic stage to find genes related to the apomeiotic pathway. We also found that apomeiosis in sporophytic thalli produce mt^±^ thalli, which release diploid biflagellate zoids. Although there is a morphological and behavioral difference between the diploid biflagellate zoids and the biflagellate zoosporoids released from the asexual thalli (e.g., the existence of the mating structure and conjugation ability), asexual populations of *U*. *prolifera* most likely originated from this apomeiotic pathway in sporophytic thalli. Moreover, the distribution of the *U*. *prolifera* population in the Shimanto River^37^ suggests that the sexual and asexual populations have different environmental tolerances of stress factors, such as salinity, nutrient levels, and temperature. In addition to molecular biological analysis for uncovering the genetic basis of apomixis, ecological research will help clarify how sexual and asexual reproduction in *U*. *prolifera* is involved in environmental adaptation.

## Methods

### Culture conditions

Table 1 summarizes strain information. Although the *U*. *prolifera* MT has previously been described as male or female based on the slight tendency toward anisogamy^11^, we used the term ‘mt^−^’ for male and ‘mt^+^’ for female, consistent with the usage in a previous study in *U*. *partita*^22,27^. Unialgal cultures were established from meiospores, gametes, or zoosporoids using the induction method^38^. Released meiospores, gametes, or zoosporoids were cultured in petri dishes at 20 °C with a 14:10-h light-dark (LD) cycle under fluorescent light at 100 µmol m^−2^ s^−1^ in artificial seawater (ASW; MARINE ART SF-1, Tomita Pharmaceutical Co., Ltd., Tokushima, Japan) supplemented with PES medium stock solution^39^. Upon reaching a size of 1 cm, some thalli were placed in 1-L aerated flasks and cultured for 2–3 weeks.

### Genotyping of field materials and F1 progeny of *U*. *prolifera* using MT-locus genes

To isolate orthologs of the MT-locus genes of *U*. *partita* in *U*. *prolifera*^22^, all the MT-locus genes of *U*. *partita* were queried against contigs derived from assembled RNA-Seq data of the mt^−^ and mt^+^ gametes and the asexual biflagellate and quadriflagellate zoosporoids of *U*. *prolifera* using the TBLASTX program. *UpRWP1* (mt^−^) and *Up0832* (mt^+^)^22^ were used as MT markers among the MT-locus gene orthologs found in the contigs of *U*. *prolifera*. A gametolog, *UpPRA*^22^, which was used for molecular phylogenetic analysis in *Ulva* to indicate the conserved differentiation of the MT locus in the genus, has also been used as an MT marker. To identify the genotype of individual strains using the MT-locus genes, DNA was extracted from each culture strain and from thalli developed from meiospores released by a single sporophyte (Up01 × Up02) using the DNeasy Plant Mini Kit (Qiagen, Valencia, CA, USA) or CicaGeneus DNA Extraction Regents ST (Kanto Chemical Co., Inc., Tokyo, Japan). The primer sequences are listed in Supplementary Table 10. To amplify each of the marker fragments, the Kapa Taq PCR Kit (Kapa Biosystems, Woburn, MA, USA) was used following the manufacturer’s protocol. The PCR program for each gene consisted of an initial denaturation step of 3 min at 95 °C, followed by 45 cycles of denaturation for 30 s at 94 °C, annealing for 30 s at 58 °C, and extension for 90 s at 68 °C. The obtained DNA fragments were separated by electrophoresis and visualized by staining with GelRed (Biotium, Fremont, CA, USA).

### Measurement of the relative amounts of DNA

To compare the relative amounts of DNA, the DNA content of each gamete and asexual zoosporoid was measured. Gamete and zoosporoid formation were induced by the punching method^38^. Gamete and asexual zoosporoids were collected using positive or negative phototaxis and fixed with 1% glutaraldehyde (GA) in ASW at 4 °C for 2 hr. After fixation, the cells were washed with PBS three times. Each cell was stained with 0.5 μg/L DAPI diluted with PBS for 10 min at room temperature. After washing with PBS, the cells were observed with a fluorescence microscope (DM6000B; Leica Microsystems, Wetzlar, Germany). The fluorescence intensity was measured using ImageJ software and calibrated using InSpeck™ Blue (350/440) Microscope Image Intensity Calibration Kit (Thermo Fisher, Waltham, MA, USA).

### Observation of the ultrastructure of gametes and zoosporoids by FE-SEM

Meiospores, gametes, and zoosporoids were prepared using the induction procedure^38^. Individual gametes, zoosporoids, and mt^±^ biflagellate zoids were fixed using a previously described methodology^23^. Crossings between gametes and biflagellate zoids released from the F1-14 strain were performed at room temperature. The cells were fixed^23^ 2 min after mixing. Post-fixation was for 2 h at 4 °C in 1% OsO_4_ dissolved in 0.05 M phosphate buffer (pH 7.4). After post-fixation, samples were treated in 1% tannic acid dissolved in 0.05 M phosphate buffer (pH 7.4) for 15 min at room temperature, and then fixed for 2 h at 4 °C in 1% OsO_4_ dissolved in 0.05 M phosphate buffer (pH 7.4). The samples were dehydrated through a graded series of ethanol solutions, infiltrated with t-butyl alcohol and freeze-dried (ES-2030; Hitachi, Tokyo, Japan). Samples were coated with platinum–palladium using ion sputter (E1045; Hitachi Corp., Tokyo, Japan). Observations were made under a FE-SEM (JSM6330F; JEOL Ltd., Tokyo, Japan) at 5 kV.

### Chromosome analysis

Thalli containing the cells in meiotic phase were collected at 30–32 h after the induction of gametogenesis or sporogenesis. Mitosis of *Ulva* occurs during the dark period^40^. Therefore, each thallus was collected during the dark period of the LD cycles. Thalli were fixed in 1% GA in ASW at 4 °C for 2 h, and then washed with PBS three times. Thalli were treated with an enzyme mixture^41^ at 30 °C for 3 h to digest their cell walls. Thalli were washed with PBS three times and stained with SYBR Green I. Chromosomes were observed using a fluorescence microscope (DM6000B; Leica Microsystems). Florescence images of SYBR Green I were captured by a CCD camera (DFC360 FX; Leica Microsystems) as 0.5-μm-thick stacks in the z-axis and each image was deconvoluted by LAS AF ver. 2.6.0 software (Leica Microsystems).

### RNA-Seq analyses

We conducted transcriptome analysis to clarify the molecular basis of the morphological and biological differences between the mt^−^ and mt^+^ gametes and asexual biflagellate and quadriflagellate zoosporoids. Total RNA was extracted from mt^−^ and mt^+^ gametes and asexual biflagellate and quadriflagellate zoosporoids using the RNeasy Plant Mini Kit (Qiagen, Valencia, CA, USA). Contaminating DNA was removed using RNase-Free DNase I (Qiagen). cDNA library construction and sequencing with the Illumina Hiseq. 2500 platform (Illumina, San Diego, CA, USA) were carried out by BGI (Shenzhen, China). The short-reads data were merged and then assembled using Trinity software^42^. The contigs obtained were clustered using Cd-hit software^43^; homologous genes located in the MT locus of *U*. *partita*^22^ and related to meiosis in eukaryotes^44,45^ were identified by BLAST X from the database for *U*. *prolifera* clustered contigs. Each of the short reads was mapped onto each of the clustered contigs using Bowtie software^46^, and the FPKM value of each contig was calculated by eXpress^47^ software on the Maser (Management and Analysis System for Enormous Reads) platform (https://cell-innovation.nig.ac.jp). Calculation of z-scores and clustering analysis based on the FPKM values for each gene were carried out using the gplots (2.16.0) package with R software (3.5.1; http://www.R-project.org/). Statistical testing for gene expression was performed in R with DESeq2 (3.8) using the no replicate method^48^. A Wilcoxon signed-rank test was conducted using Python 3.6.1/SciPy 0.19.1 software to assess the statistical significance between the gene expression patterns of the mt^−^ and mt^+^ gametes and asexual biflagellate and quadriflagellate zoosporoids.

## Supplementary information

Supplementary Information
