# Supplementary material for: Asexual thalli originated from sporophytic thalli via apomeiosis in the green seaweed Ulva
Source: Sci Rep. 2019 Sep 18;9:13523. doi: 10.1038/s41598-019-50070-x (PMC6751216; doi:10.1038/s41598-019-50070-x)
Supplement: Supplementary file 1 — Supplementary Information [file 41598_2019_50070_MOESM1_ESM.pdf]

Title

Asexual thalli originated from sporophytic thalli via apomeiosis in the green seaweed *Ulva*

Authors

Kensuke Ichihara, Tomokazu Yamazaki, Shinichi Miyamura, Masanori Hiraoka and Shigeyuki Kawano

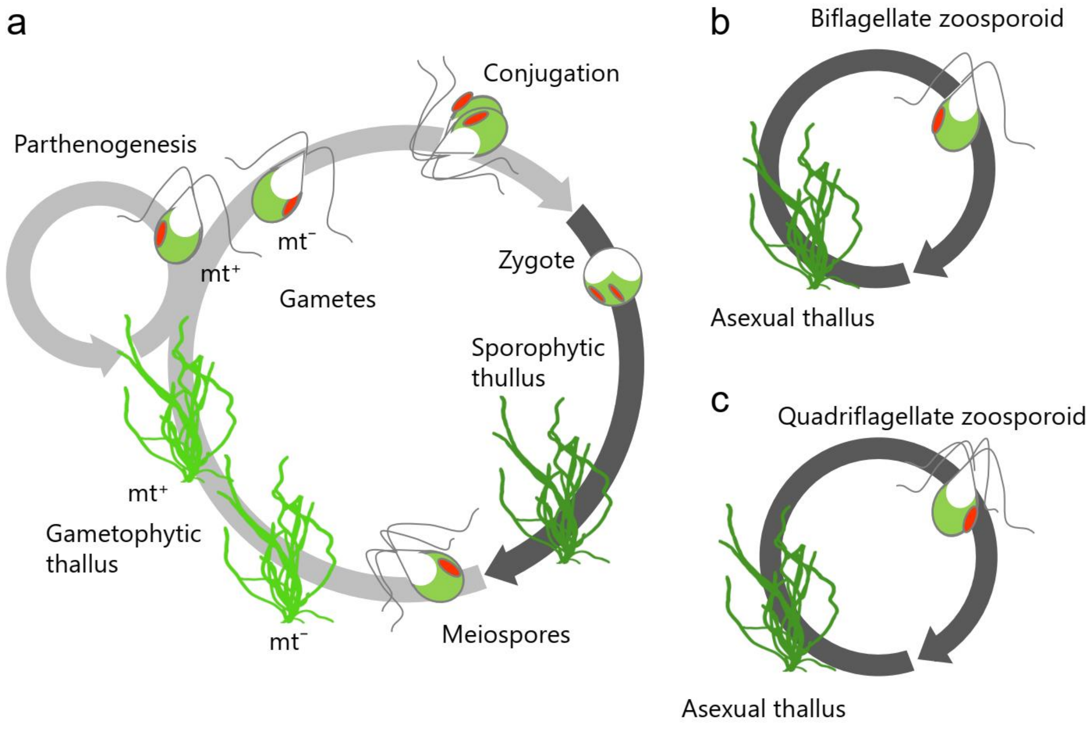

Supplementary Fig. 1 Three different life cycle of *U. prolifera*. (a) sexual life cycle, (b) asexual life cycle via a biflagellate zoosporoid, (c) asexual life cycle via a quadriflagellate zoosporoid. Grey line: Haploid phase, dark grey line: diploid phase. Light green: Gametophytic thallus (N), Dark green: Sporophytic or asexual thallus (2N).

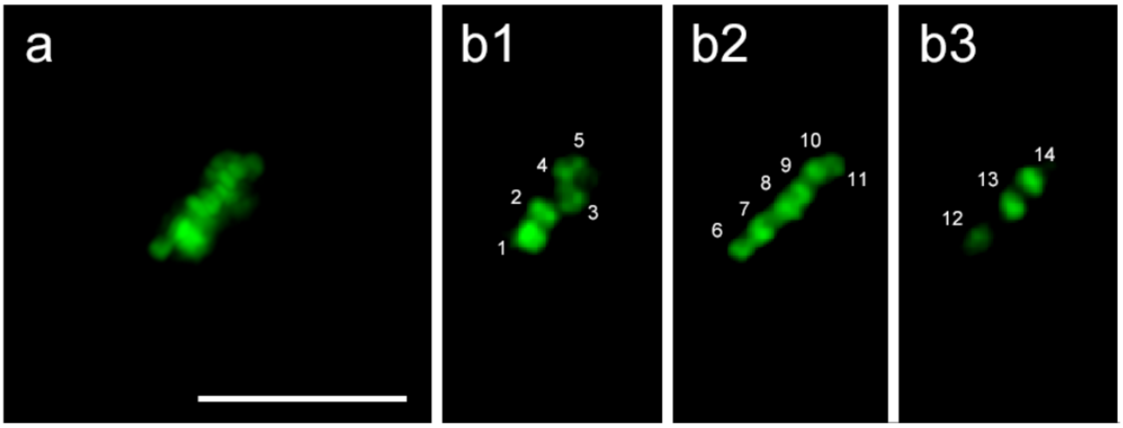

Supplementary Fig. 2. Chromosomes in an asexual thallus via quadriflagellate zoosporoid during mitotic mid-metaphase. (a) the stacked image of chromosomes. (b1-3) Optical sections for counting chromosomes. Scale bar, 7.5 μm.

a

	uncoupled cells	zygotes
Biflagellate zoids x mt^- gametes	108	55
Biflagellate zoids x mt^+ gametes	150	0

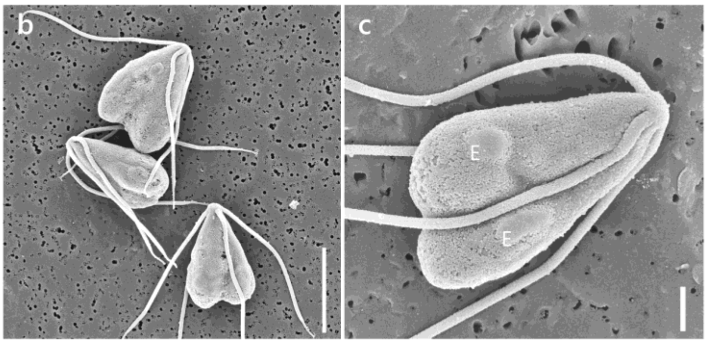

Supplementary Fig. 3. Cross experiment between the biflagellate zoids released from $mt^{\pm}$ thallus (F1-14) and mt^- or mt^+ gametes. (a) zygote number between the biflagellate zoids and each gamete. (b, c) Zygote between biflagellate zoids and mt^- gamete. E, eyespot. Scale bar, 5 μm (b), 1 μm (c).

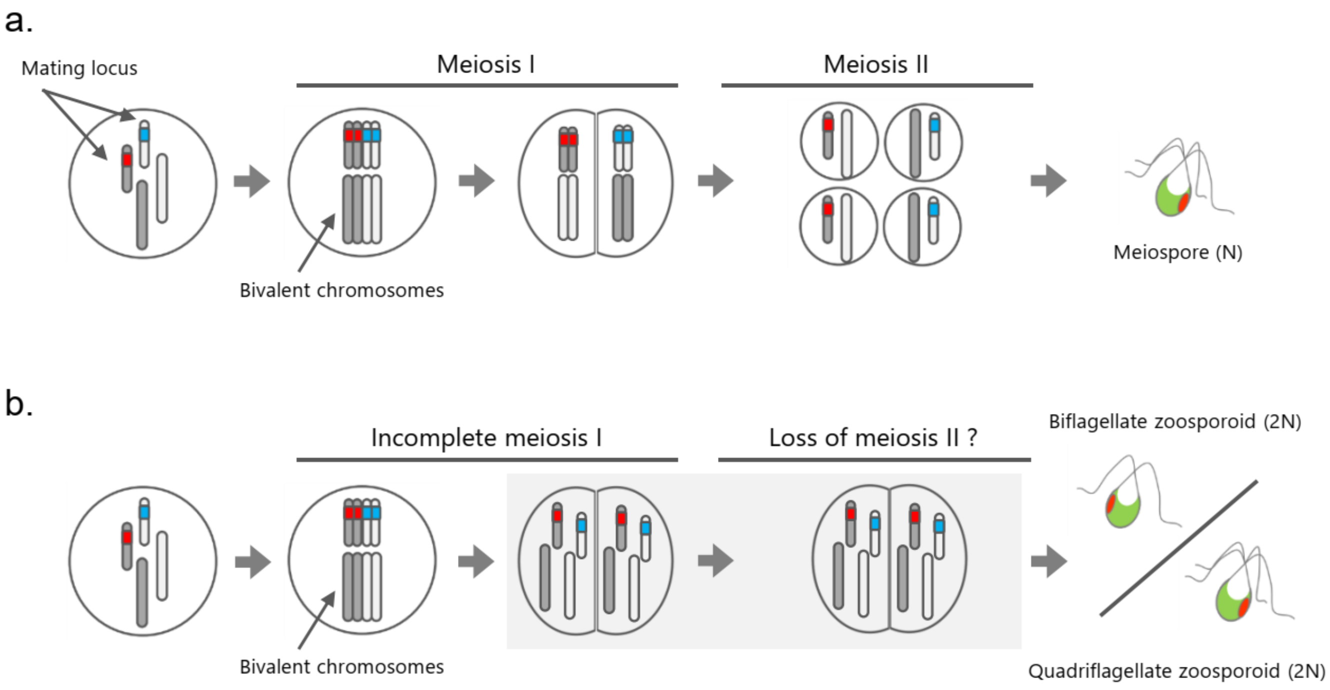

Supplementary Fig. 4. Schematic model of chromosomal dynamics during formation of each reproductive cell in *U. prolifera*. (a) Major part of cells in sporophytic thalli. (b) Cells in asexual thalli (biflagellate type and quadriflagellate type) and minor part of cells in sporophytic thalli.

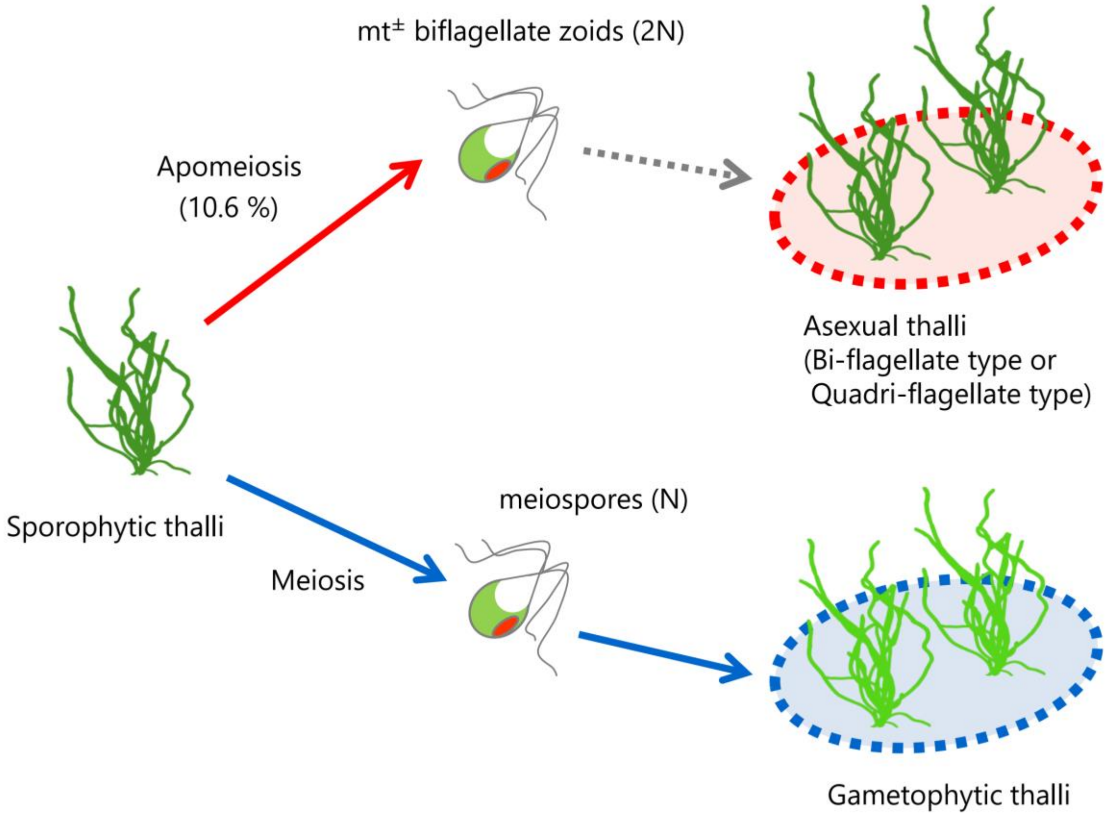

Supplementary Fig. 5. Schematic model of the emerging pathway of asexual thalli in *U. prolifera*. Normal meiosis occurred in a major part of cells in the sporophytic thalli, but a minor part of cells carried out apomeiosis without the reduction of chromosomal numbers. Light green: Gametophytic thallus (N), Dark green: Sporophytic or asexual thallus (2N).

Supplementary Table 1. Summary of RNA-seq data

Sample	Clean Reads	Clean bases	Q20 (%)	GC (%)	Accession
Mt^- gamete (Up01)	33,277,028	3,327,702,800	98.95;98.56	59.86	DRA008092
Mt^+ gamete (Up02)	32,726,812	3,272,681,200	98.92;98.60	59.72	DRA008093
Biflagellate zoosporoid (Up13)	33,200,888	3,320,088,800	98.93;98.47	59.76	DRA008095
Quadriflagellate zoosporoid (Up17)	32,243,352	3,224,335,200	98.93;98.42	60.31	DRA008098

Supplementary Table 2. Homologous gene of *U. partita*'s gametologs

gene name	Mating type minus			Mating type plus		
	Accession No. of *U. partita*	*U. prolifera* contig No.	Accession No.	Accession No. of *U. partita*	*U. prolifera* contig No.	Accession No.
ALP1	LC088508	Contig24538	ICLF01000045	LC088590	Contig23506	ICLF01000001
SLG1	LC088561	Contig9414	ICLF01000046	LC088606	Contig42876	ICLF01000002
01506	LC088578	Contig26331	ICLF01000047	LC088597	Contig45177	ICLF01000003
PIK1	LC088571	Contig26388	ICLF01000048	LC088632	Contig47971	ICLF01000004
SOM1	LC088530	Contig8000	ICLF01000050	LC088652	Contig6137	ICLF01000006
03244	LC088521	Contig47702	ICLF01000051	LC088623	Contig47699	ICLF01000007
06774	LC088520	Contig22873	ICLF01000052	LC088661	Contig45122	ICLF01000008
PRA1	LC088563	Contig21844	ICLF01000009	LC088633	Contig21844	ICLF01000009
PRP1	LC088502	Contig4526	ICLF01000053	LC088626	Contig5106	ICLF01000010
SNR1	LC088560	Contig14623	ICLF01000054	LC088608	Contig43254	ICLF01000011
HAR1	LC088504	Contig49321	ICLF01000012	LC088671	Contig49321	ICLF01000012
03265	LC088501	Contig11547	ICLF01000014	LC088620	Contig11547	ICLF01000014
12223	LC088515	Contig4909	ICLF01000056	LC088600	Contig46730	ICLF01000015
12186	LC088577	Contig24120	ICLF01000057	LC088593	Contig5112	ICLF01000016
ACTB1	LC088577	Contig45520	ICLF01000058	LC088670	Contig45519	ICLF01000017
CKK-CNB1	LC088549	Contig7731	ICLF01000059	LC088639	Contig6080	ICLF01000018
DGK1	LC088523	Contig23952	ICLF01000060	LC088595	Contig34081	ICLF01000019
eIF1	LC088532	Contig14392	ICLF01000061	LC088588	Contig42652	ICLF01000020
LOG1	LC088517	Contig7766	ICLF01000062	LC088634	Contig7767	ICLF01000021
MAPKKK1	LC088555	Contig26873	ICLF01000022	LC088664	Contig26873	ICLF01000022
MET1	LC088534	Contig33210	ICLF01000063	LC088647	Contig43065	ICLF01000023

Supplementary Table 3. Homologous gene of *U. partita*'s mating type specific genes

Mating type minus				Mating type plus			
gene name	Accession No. of *U. partita*	*U. prolifera* contig No.	Accession No.	gene name	Accession No. of *U. partita*	*U. prolifera* contig No.	Accession No.
01839m	LC088524	Contig5228	ICLF01000064	*02227f*	LC088611	Contig49462	ICLF01000024
GTB1m	LC088546	Contig42872	ICLF01000065	*02393f*	LC088599	Contig45177	ICLF01000003
05930m	LC088566	Contig4474	ICLF01000066	*07255f*	LC088668	Contig14538	ICLF01000025
07231m	LC088540	Contig49699	ICLF01000067	*00368f*	LC088637	Contig7764	ICLF01000026
03836m	LC088542	Contig42392	ICLF01000068	*06379f*	LC088678	Contig11547	ICLF01000014
06406m	LC088516	Contig26873	ICLF01000022	*03244f*	LC088623	Contig9461	ICLF01000028
06778m	LC088541	Contig6607	ICLF01000069	*04234f*	LC088645	Contig16978	ICLF01000029
03057m	LC088556	Contig46045	ICLF01000070	*03694f*	LC088679	Contig16907	ICLF01000030
07600m	LC088544	Contig11722	ICLF01000071	*02423f*	LC088676	Contig4466	ICLF01000031
SLG1m	LC088561	Contig9415	ICLF01000072	*SOM1f*	LC088652	Contig25603	ICLF01000033
06021m	LC088584	Contig29738	ICLF01000073	*05479f*	LC088680	Contig42540	ICLF01000034
07727m	LC088545	Contig11723	ICLF01000074	*07113f*	LC088622	Contig50740	ICLF01000035
RWP1	LC088585	Contig6606	ICLF01000075	*07287f*	LC088651	Contig46731	ICLF01000036
07877m	LC088569	Contig47546	ICLF01000076	*07525f*	LC088621	Contig18933	ICLF01000037
				07334f	LC088609	Contig45990	ICLF01000038
				03910f	LC088629	Contig12594	ICLF01000039
				00345f	LC088628	Contig6738	ICLF01000040
				03772f	LC088646	Contig36363	ICLF01000041
				03786f	LC088663	Contig40902	ICLF01000042
				06478f	LC088644	Contig38281	ICLF01000043

Supplementary Table 4. p values calculated by Wilcoxon signed-rank test in expression pattern of mt^- gametologs.

	mt^- gamete	mt^+ gamete	biflagellate zoosporoid	quadriflagellate zoosporoid
mt^- gamete		0.00331*	0.00461*	0.20456
mt^+ gamete	0.00331*		0.01174*	0.00331*
biflagellate zoosporoid	0.00461*	0.01174*		0.35701
quadriflagellate zoosporoid	0.20456	0.00331*	0.35701	

(*$p < 0.05$)

Supplementary Table 5. p values calculated by Wilcoxon signed-rank test in expression pattern of mt^+ gametologs.

	mt^- gamete	mt^+ gamete	biflagellate zoosporoid	quadriflagellate zoosporoid
mt^- gamete		0.00784*	0.10747	0.11654
mt^+ gamete	0.00784*		0.00370*	0.28910
biflagellate zoosporoid	0.10747	0.00370*		0.11654
quadriflagellate zoosporoid	0.11654	0.28910	0.11654	

(*$p < 0.05$)

Supplementary Table 6. p values calculated by Wilcoxon signed-rank test in expression pattern of mt^- specific genes.

	mt^- gamete	mt^+ gamete	biflagellate zoosporoid	quadriflagellate zoosporoid
mt^- gamete		0.63777	0.59362	0.14015
mt^+ gamete	0.63777		0.20943	0.50675
biflagellate zoosporoid	0.59362	0.20943		0.31090
quadriflagellate zoosporoid	0.14015	0.50675	0.31090	

(*$p < 0.05$)

Supplementary Table 7. p values calculated by Wilcoxon signed-rank test in expression pattern of mt^{+} specific genes.

	mt^{-} gamete	mt^{+} gamete	biflagellate zoosporoid	quadriflagellate zoosporoid
mt^{-} gamete		0.01988*	0.08539	0.00842*
mt^{+} gamete	0.01988*		0.10747	0.64747
biflagellate zoosporoid	0.08539	0.10747		0.01113*
quadriflagellate zoosporoid	0.00842*	0.64747	0.01113*	

(*$p < 0.05$)

Supplementary Table 8. Meiosis related genes in *U. prolifera*

Meiotic Stage	gene name	Accession No.	organism	e value	*U. prolifera* contig	Accession No.
1. Entry into meiosis	*AGO9*	NP_197613.2	*Arabidopsis thaliana*	4.00E-71	Contig37408	ICLF01000077
	AGO5	XP_008673577.1	*Zea mays*	2.00E-30	Contig11441	ICLF01000078
	MEI1	NP_177856.2	*Arabidopsis thaliana*	1.00E-13	Contig45509	ICLF01000079
	Mei2L	Q6ZI17	*Oryza sativa* subsp. *japonica*	5.00E-33	Contig13495	ICLF01000080
2. Recombination	*CRC1/Pch2*	XP_010102684.1	*Morus notabilis*	9.00E-98	Contig45203	ICLF01000081
	ATM/Tel1	AT3G48190	*Arabidopsis thaliana*	1.00E-43	Contig50683	ICLF01000082
	Com1/Sae2	AT3G52115	*Arabidopsis thaliana*	2.80E-02	Contig51894	ICLF01000083
	MCM8	AT3G09660	*Arabidopsis thaliana*	6.00E-29	Contig21685	ICLF01000084
	Mre11	AT5G54260	*Arabidopsis thaliana*	1.00E-119	Contig45844	ICLF01000085
	RAD1	XP_008676499	*Zea mays*	3.00E-82	Contig11325	ICLF01000086
	RAD51	NP_568402.1	*Arabidopsis thaliana*	3.00E-42	Contig3397	ICLF01000087
	RAD51B	AT2G28560	*Arabidopsis thaliana*	8.00E-07	Contig47936	ICLF01000088
	Rpa1	AT2G06510	*Arabidopsis thaliana*	2.00E-70	Contig7017	ICLF01000089
	Rpa2	AT2G24490	*Arabidopsis thaliana*	4.00E-10	Contig3214	ICLF01000090
	HOP2	AT1G13330	*Arabidopsis thaliana*	2.00E-08	Contig40414	ICLF01000091
	MLH1	AT4G09140	*Arabidopsis thaliana*	7.00E-37	Contig45061	ICLF01000092
	MSH5	NP_188683.3	*Arabidopsis thaliana*	2.00E-84	Contig48596	ICLF01000093
	MSH6	O04716.2	*Arabidopsis thaliana*	4.00E-141	Contig6450	ICLF01000094
	RECQ4A	NP_172562.2	*Arabidopsis thaliana*	1.00E-119	Contig44108	ICLF01000095
	RFC1	NP_680188.1	*Arabidopsis thaliana*	8.00E-28	Contig53156	ICLF01000096
	AXR1	P42744.1	*Arabidopsis thaliana*	2.00E-10	Contig12181	ICLF01000097
	PSS1/Kinesin 1	XP_006598200.1	*Glycine max*	7.00E-68	Contig23091	ICLF01000098
3. Sister chromatid	*Cnd1*	Q8K2Z4	*Mus musculus*	7.00E-17	Contig45869	ICLF01000099
cohesion	*Eso1*	NP_596778.1	*Schizosaccharomyces pombe*	9.00E-48	Contig44586	ICLF01000100
	REC11 (SA3)	NP_588108.3	*Schizosaccharomyces pombe*	5.00E-04	Contig23090	ICLF01000101
	SA1	XP_011510631.1	*Homo sapiens*	6.00E-39	Contig30826	ICLF01000102
	Scc2/Mis4	XP_003618719.2	*Medicago truncatula*	1.00E-09	Contig29652	ICLF01000103
	SCC3	O82265.2	*Arabidopsis thaliana*	7.00E-21	Contig34877	ICLF01000104
	SMC1	Q6Q1P4.2	*Arabidopsis thaliana*	5.00E-140	Contig8515	ICLF01000105
	SMC3	NP_001190492	*Arabidopsis thaliana*	4.00E-84	Contig11349	ICLF01000106
	SYN3	NP_851110.1	*Arabidopsis thaliana*	9.00E-09	Contig42706	ICLF01000107

4. Cell cycle control: spindle–cytokinesis	*CDKA;1*	AT3G48750	*Arabidopsis thaliana*	2.00E-77	Contig34927	ICLF01000108
	Kinesin 14	AT4G21270	*Arabidopsis thaliana*	1.00E-74	Contig45500	ICLF01000109
	Kinesin 7	AT3G43210	*Arabidopsis thaliana*	7.00E-69	Contig37961	ICLF01000110
	Skp1	AT1G75950	*Arabidopsis thaliana*	3.00E-47	Contig28152	ICLF01000111
	TAM	NP_177863.2	*Arabidopsis thaliana*	1.00E-45	Contig42403	ICLF01000112

Supplementary Table 9. p values calculated by Wilcoxon signed-rank test in expression pattern of meiosis related genes.

	mt^- gamete	mt^+ gamete	biflagellate zoosporoid	quadriflagellate zoosporoid
mt^- gamete		0.81863	0.19008	0.10490
mt^+ gamete	0.81863		0.11378	0.03778*
biflagellate zoosporoid	0.19008	0.11378		0.99287
quadriflagellate zoosporoid	0.10490	0.03778*	0.99287	

(*p < 0.05)

Supplementary Table 10. Primers list for MT locus genotyping.

	Primer name	sequences
	UpRWP1_F	CGCAAGCGCTGATGATTGAA
mt^- specific	UpRWP1_R	CCACGACGAAACTGTCTCCA
primers	M_PRAm_527F	CAGAGCCACTGCAGAAAATT
	M_PRAm_1055R	GGCTGCAAGGACTGAAGAGG
	Up0832_F	CGGCATGTGTTCCAAAGCAA
mt^+ specific	Up0832_R	TTGCTGTCAGGAATGAGCGT
primers	F_PRAf_103F	GCCGCAGATGTGTGCAATCA
	F_PRAf_430R	ACAGGAGTCTCGTCAGATCC
